# Territorywide Study of Early Coronavirus Disease Outbreak, Hong Kong, China 

**DOI:** 10.3201/eid2701.201543

**Published:** 2021-01

**Authors:** Kenneth Siu-Sing Leung, Timothy Ting-Leung Ng, Alan Ka-Lun Wu, Miranda Chong-Yee Yau, Hiu-Yin Lao, Ming-Pan Choi, Kingsley King-Gee Tam, Lam-Kwong Lee, Barry Kin-Chung Wong, Alex Yat Man Ho, Kam-Tong Yip, Kwok-Cheung Lung, Raymond Wai-To Liu, Eugene Yuk-Keung Tso, Wai-Shing Leung, Man-Chun Chan, Yuk-Yung Ng, Kit-Man Sin, Kitty Sau-Chun Fung, Sandy Ka-Yee Chau, Wing-Kin To, Tak-Lun Que, David Ho-Keung Shum, Shea Ping Yip, Wing Cheong Yam, Gilman Kit-Hang Siu

**Affiliations:** The University of Hong Kong, Hong Kong, China (K.S.-S. Leung, K.K.-G. Tam, W.C. Yam);; The Hong Kong Polytechnic University, Hong Kong (T.T.-L. Ng, H.-Y. Lao, M.-P. Choi, L.-K. Lee, D.H.-K. Shum, S.P. Yip, G.K.-H. Siu);; Pamela Youde Nethersole Eastern Hospital, Hong Kong (A.K.-L. Wu, M.C.-Y. Yau, K.-C. Lung);; United Christian Hospital, Hong Kong (B.K.-C. Wong, E.Y.-K. Tso, K.S.-C. Fung, S.K.-Y. Chau);; Princess Margaret Hospital, Hong Kong (A.Y.-M. Ho, W.-S. Leung, M.-C. Chan, W.-K. To);; Tuen Mun Hospital, Hong Kong (K.-T. Yip, Y.-Y. Ng, K.-M. Sin, T.-L. Que); Ruttonjee Hospital, Hong Kong (R.W.-T. Liu)

**Keywords:** novel coronavirus, COVID-19, SARS-CoV-2, severe acute respiratory syndrome coronavirus 2, viruses, respiratory infections, community outbreak, whole genome sequencing, clinical demographic, molecular phylogeny, molecular evolutionary analysis, Hong Kong, China

## Abstract

Initial cases of coronavirus disease in Hong Kong were imported from mainland China. A dramatic increase in case numbers was seen in February 2020. Most case-patients had no recent travel history, suggesting the presence of transmission chains in the local community. We collected demographic, clinical, and epidemiologic data from 50 patients, who accounted for 53.8% of total reported case-patients as of February 28, 2020. We performed whole-genome sequencing to determine phylogenetic relationship and transmission dynamics of severe acute respiratory syndrome coronavirus 2 infections. By using phylogenetic analysis, we attributed the community outbreak to 2 lineages; 1 harbored a common mutation, *Orf3a-*G251V, and accounted for 88.0% of the cases in our study. The estimated time to the most recent common ancestor of local coronavirus disease outbreak was December 24, 2019, with an evolutionary rate of 3.04 × 10^−3^ substitutions/site/year. The reproduction number was 1.84, indicating ongoing community spread.

Coronavirus disease (COVID-19) refers to a cluster of viral pneumonia cases that first occurred in Wuhan, a city in Hubei Province, China, beginning in December 2019. Etiology was unknown during the early stage of the outbreak until a novel coronavirus, severe acute respiratory syndrome coronavirus 2 (SARS-CoV-2), was isolated on January 7, 2020, and the genome was sequenced (*1*).

During the initial outbreak, fever was the main symptom of COVID-19, and about one third of patients experienced acute respiratory distress syndrome. Approximately 16% of patients were in severe condition at admission, and the estimated mortality rate was 1.4% (*2*). Sustained human-to-human transmission was confirmed upon identification of cases clustering among families and transmission from patients to healthcare workers (*3*,*4*), which triggered China’s urgent public health actions and international concern.

As of February 28, 2020, a total of 78,824 COVID-19 cases had been diagnosed in mainland China and 2,788 persons had died. The disease had also spread to 50 other countries (*5*). The World Health Organization declared COVID-19 a pandemic in March 2020. 

In Hong Kong, the first imported case was identified on January 23, 2020; the case-patient was a resident of mainland China who traveled to Hong Kong from Wuhan through Shenzhen by high-speed rail. The first local case with unknown source (i.e., patient who had no travel record during the 14-day incubation period) was reported on February 4, 2020 (*6*).

By February 28, 2020, a total of 93 COVID-19 cases had been recorded in Hong Kong; >70 (75.3%) of those were local cases and those case-patients’ close contacts (*6*,*7*). Secondary and tertiary transmissions were observed in some case clusters. Because source of infection is unknown in most index cases in these clusters, hidden transmission chains were believed to be present in the community.

We report the demographic, clinical, and epidemiologic data of 50 hospitalized patients who accounted for 53.8% of COVID-19 cases in Hong Kong at the data cutoff point (February 28, 2020), including 3 imported cases and 6 transmission clusters of local infections. We characterized viral genomes in all these cases by using nanopore and Illumina sequencing. Phylogenetic and molecular evolutionary analyses were performed to determine the transmission link and the evolutionary rate of COVID-19 cases in the community.

## Methods

### Cases

For this retrospective, multicenter study, we enrolled case-patients with laboratory-confirmed COVID-19 from 4 public hospital clusters managed under the Hospital Authority of Hong Kong, namely Hong Kong East Cluster, Kowloon East Cluster, Kowloon West Cluster, and New Territories West Cluster, during January 26–February 28, 2020. Sputum specimens and throat swab specimens pooled with nasopharyngeal aspirates were collected from patients who fulfilled the reporting or enhanced surveillance criteria at hospital admission (*8*). Laboratory-confirmed infection was defined as the detection of SARS-CoV-2 by real-time reverse transcription PCR, which amplified the envelope (*E*) gene and RNA-dependent RNA polymerase (*RdRp*) gene (*9*).

We obtained demographic, clinical, and microbiologic data from patients’ medical records. Epidemiologic information was retrieved from the Centre for Health Protection of the Department of Health (*6*) and the website https://wars.vote4.hk (*7*). The definitions of clinical symptoms and complications are based on World Health Organization guidance (*10*). We adopted the Centre for Health Protection case numbering system, which is based on the date of case confirmation. This study was approved by the Institutional Review Boards of The Hong Kong Polytechnic University (approval no. RSA20021) and the public hospitals involved (HKECREC-20200014; KCC/KEC-20200070; KWC-20200040; NTWC-20200038).

### Specimen Preparation

The respiratory specimens were centrifuged at 16,000 × *g* for 2 minutes. Total nucleic acid was extracted from supernatant using MagNA Pure 96 System (Roche, https://lifescience.roche.com) or NucliSENS easyMAG (bioMérieux, https://www.biomerieux-nordic.com) according to the manufacturers’ instructions. DNase treatment was done by using TURBO DNA-free Kit (ThermoFisher Scientific, https://www.thermofisher.com) to remove residual host DNA.

### Reverse Transcription and Viral Genome Amplification Using Multiplex PCR

DNase-treated RNA was reverse-transcribed using random hexamers and SuperScript IV Reverse Transcriptase (ThermoFisher Scientific) as previously described (*11*). Viral cDNA was then amplified by using 2 PCRs containing tiled, multiplexed primers (Appendix 1 Table 1) described in the ARTIC protocol (https://artic.network/ncov-2019) (*12*). Details of the multiplex PCR are provided in Appendix 2.

### Nanopore MinION Sequencing

Ligation-based 1D sequencing was carried out by using Litigation Sequencing Kit SQK-LSK109 (Oxford Nanopore Technologies, https://nanoporetech.com) according to manufacturer’s instructions. Multiplex PCR amplicons of each sample were normalized to 1 ng/µL before end-repair and native barcode ligation by using EXP-NBD104/114 (Oxford Nanopore Technologies). Barcoded samples were pooled and ligated to AMII sequencing adaptor. Sequencing was performed with Nanopore MinION device (Oxford Nanopore Technologies) by using R9.4.1 flow cell for 48 hours.

### Illumina MiSeq Sequencing

Multiplex PCR amplicons were subjected to library preparation and dual-indexing by using KAPA HyperPrep Kit and Unique Dual-Indexed Adaptor Kit (Roche) according to manufacturer’s instructions. Ligated libraries were enriched by 6-cycle PCR amplification and purification and size selection by using AMPure XP beads (Beckman Coulter, https://www.beckmancoulter.com/). The pooled library was sequenced with the MiSeq Reagent Kit V2 Nano on an Illumina MiSeq System (Illumina, https://www.illumina.com).

### Bioinformatic Analysis

We analyzed nanopore sequencing data using modified Artic Network nCoV-2019 novel coronavirus bioinformatics protocol (Appendix 2) (*13*). Illumina sequencing reads were mapped with reference to respective consensus genome of each sample constructed from nanopore data. Variants were called by using freebayes version 1.0.0 (https://github.com/freebayes/freebayes) with haploid decoding and minimum base quality set at Q30. Consensus genomes were constructed by GATK 4.1.4.1 based on the VCF file (*14*). SPAdes genome assembler 3.14.0 (https://cab.spbu.ru/software/spades) and minimap2 version 2.17 (https://anaconda.org/bioconda/minimap2) were used to combine nanopore and Illumina sequencing results for de novo assembly and to identify the sequence of the unmapped gap regions. The sequences have been submitted to GenBank (accession nos. MT232662711).

### Genomic and Phylogenetic Analysis

To identify the amino acid change caused by each single-nucleotide polymorphism, we BLAST-searched the consensus genome of each specimen against the reference NC_045512.2 using BLASTX (https://blast.ncbi.nlm.nih.gov/Blast.cgi). Nonsynonymous mutations were identified using custom Python script (https://github.com/kenssl/Blast_mismatch_search).

Consensus genomes were aligned by Clustal Omega 1.2.4 (https://www.ebi.ac.uk/Tools/msa/clustalo). Phylogenetic tree was constructed with PhyML 3.0 (http://www.atgc-montpellier.fr/phyml/) using the maximum-likelihood algorithm. Best-fitting substitution model was selected by Akaike information criteria, in which we selected the general time-reversible model with fixed proportion of invariable sites (*15*). Bootstrap replicates were set at 1,000×, and maximum-likelihood phylogenetic tree was rooted on the earliest published genome (accession no. NC_045512.2). Transmission clusters were defined by clear epidemiologic and onset-time relationship. Meanwhile, we downloaded an additional 478 SARS-CoV-2 genomes from the GISAID (https://www.gisaid.org) SARS-CoV-2 data hub (*16*) and analyzed the phylogenetic relationships by using maximum-likelihood with bootstrap value set at 500× and rooted on SARS-CoV-2 genome NC_045512.2.

### Estimation of Evolutionary Rate and Divergence Time of Transmission

To reconstruct the evolutionary model of COVID-19 cases using the viral genomes obtained in Hong Kong, we implemented Bayesian inference through Markov Chain Monte Carlo (MCMC) framework in BEAST version 2.6.2 (*17*). Death rate δ (which refers to the time needed for a case-patient to become noncontagious) was determined as the lag time between date of symptom onset and date of hospital admission, because the transmission link in Hong Kong was practically stopped once the patient was hospitalized. Bayesian phylodynamic analysis was performed using strict clock and relaxed clock models with coalescent exponential growth tree priors. We ran MCMC chains for 10^9^ generations and sampled every 500 steps. Bayesian output was analyzed after the results were visualized by Tracer version 1.7.1 (*18*). All parameters had an effective sample size of >200, indicating sufficient sampling.

## Results

Our investigation included 50 COVID-19 patients; 54.0% were women, and the mean age was 55.2 (range 22–96) years (Table 1). Of the case-patients, we categorized 3 cases as imported because the patients stayed in Wuhan before traveling to Hong Kong in mid-January. Four patients traveled to Japan and other provinces of China in mid-January and were hospitalized after they returned to Hong Kong, but active community transmission of COVID-19 was not officially reported in these areas during the study period, so these cases were considered possible local infections. The other 43 patients’ cases were categorized as local infection because of no recent travel history. 

**Table 1:** Demographics, travel record, and baseline medical history of 50 coronavirus disease patients, Hong Kong, February 2020*

Characteristic	Case-patients, n = 50
Age group, y	
Mean (SD)	55.2 (19.5)
Range	22–96
<30	8 (16.0)
31–40	5 (10.0)
41–50	6 (12.0)
51–60	11 (22.0)
61–70	10 (20.0)
>71	10 (20.0)
Sex	
F	27 (54.0)
M	23 (46.0)
Travel record <14 d before symptom onset	7 (14.0)
Wuhan, Hubei Province, China	3 (6.0)
Other regions in mainland China	1 (2.0)
Regions outside mainland China	3 (6.0)
No travel record	43 (86.0)
Chronic medical illnesses	18 (36.0)
Cardiovascular and cerebrovascular diseases	14 (28.0)
Endocrine system diseases	11 (22.0)
Nervous system diseases	5 (10.0)
Digestive system diseases	4 (8.0)
Malignant tumor	1 (2.0)
*Values are no. (%) except as indicated.	

Eighteen (36.0%) patients had chronic illnesses, of which cardiovascular and cerebrovascular diseases were the most common (Table 1). In total, 74.0% of the patients were experiencing cough at admission. Fever occurred in 58.0% of patients at time of admission, but that rate gradually increased to 64.0% during the course of hospitalization. Other less common symptoms were muscle aches (25.0%), sore throat (24.0%), shortness of breath (24.0%), and diarrhea (14.3%) (Table 2). Two persons (4.0%) were asymptomatic throughout the study period. On radiologic examination, 27 (54.0%) had bilateral pneumonia, 11 (22.0%) had unilateral pneumonia, and 17 (34.7%) showed multiple areas of mottling and ground-glass opacity. None of the patients were co-infected with other respiratory viruses or fungi. 

**Table 2:** Clinical characteristics and outcomes of 50 coronavirus disease patients, Hong Kong, February 2020*

Characteristic	Case-patients, n = 50*
Signs and symptoms	
Fever at admission	29 (58.0)
Fever during hospitalization	32 (64.0)
Cough	37 (74.0)
Sore throat	12 (24.0)
Shortness of breath	12 (24.0)
Muscle ache	12 (25.0)†
Diarrhea	7 (14.3)‡
Rhinorrhea	4 (8.0)
Nausea and vomiting	4 (8.2)‡
Confusion	1 (2.0)
>1 sign or symptom	41 (82.0)
Asymptomatic	2 (4.0)
Complications§	
Acute respiratory distress syndrome	2 (4.0)
Acute respiratory injury	1 (2.0)
Acute renal injury	5 (10.0)
Septic shock	1 (2.0)
>1 complication	2 (4.0)
No complications	45 (90.0)
Radiological findings	
Unilateral pneumonia	11 (22.0)
Bilateral pneumonia	27 (54.0)
Multiple areas of mottling and ground- glass opacity	17 (34.7)‡
No abnormality	4 (8.0)
Coinfection	
Other viruses	0
Bacteria	2 (4.0)¶
Fungi	0
Clinical outcome#	
In serious condition, ICU admission	3 (6.0)
Hospitalized, in stable condition	27 (54.0)
Discharged	20 (40.0)
Interval from symptom onset to hospital admission, d**	
Average (SD)	8.5 (3.9)
Range	1–26
Length of hospital stay, d††	
Average (SD)	17.7 (7.7)
Range	8–35

*Values are no. (%) except as indicated. ICU, intensive care unit. †Data were missing for 2 patients.  ‡Data were missing for 1 patient.  §Definitions of complications are provided in Appendix 2 (https://wwwnc.cdc.gov/EID/article/27/1/20-1543-App2.pdf). ¶Moderate growth of *Klebsiella aerogenes* and *Ralstonia pickettii* bacteria were obtained from sputum specimens collected from case 38 and 70. #Data cutoff of the clinical outcome of patients was February 28, 2020. **Data from symptomatic patients were excluded. ††Calculated based on the 18 patients who had been discharged as of February 28, 2020.

Intensive care unit admission was relatively uncommon (3/50, 6.0%) in our cohort compared with admission rates in previous studies (*19*–*22*). This difference could be attributed to underdiagnosis of milder cases during the initial COVID-19 outbreak in China. One patient’s sputum specimen was culture-positive for *Klebsiella aerogenes* bacteria; a second patient’s specimen was culture-positive for *Ralstonia pickettii* bacteria. Both had acute respiratory distress syndrome and acute respiratory injury accompanied by septic shock or acute renal injury and required admission to the intensive care unit.

**Figure 1:**
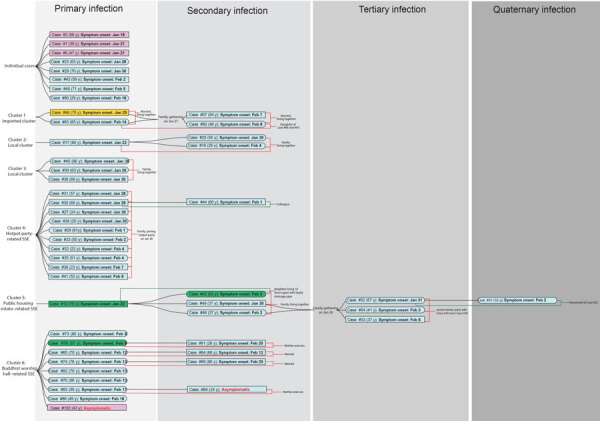
Demographics of coronavirus disease patients, Hong Kong, February 2020. Rectangular boxes indicate men or boys and stadium-shaped boxes indicate women or girls. Travel history within the 14-day incubation period before symptom onset is highlighted in cyan for local case without travel history, pink for travel history to Wuhan <14 days from symptom onset, orange for travel history to other regions in mainland China, and green for travel history to regions outside mainland China. Contact histories are highlighted by red lines for family contacts and green lines for nonfamily contacts. Case numbers are those used by the Centre of Health Protection, Department of Health, Hong Kong (*6*). Within each cluster, cases of primary infection (leftmost column) are arranged from top to bottom in order of the date (DD/MM) of symptom onset. SSE, superspreading event.

Of the 50 case-patients, 42 (84.0%) could be clustered based on their epidemiologic links (Figure 1). We identified 6 transmission clusters (clusters 1–6). Cluster 1 involved a 4-member family. The father, who traveled to Guangdong, China, in late January 2020, was believed to have infected his wife and subsequently their daughter and son-in-law at a family gathering. Clusters 2 and 3 were family clusters of local infection and unknown source. Both clusters involved 3 household members with no recent travel history. Cluster 4 was attributed to a superspreading event (SSE): a barbecue and hotpot party involving 19 family members in late January. Symptom onset in these patients occurred during days 2–13 after the party. A colleague of 1 infected person, who did not attend the party, also tested positive for SARS-CoV-2. Cluster 5 initiated from a resident of a public housing estate, in whom COVID-19 was diagnosed on January 30. Eleven days later, diagnoses were made in 3 members of a household that resided in the same building (10 stories below) the index case-patient. Two household members subsequently attended a family gathering of 29 persons at a Chinese restaurant during the incubation period. COVID-19 was diagnosed consecutively in 3 persons ≈2 weeks after the gathering. In addition, a Filipino domestic aide of 1 infected family member, who did not attend the family gathering, also tested positive. For cluster 6, the first reported case was in a 70-year-old woman who visited a Buddhist worship hall during the Chinese New Year. A further 8 persons who visited the same Buddhist worship hall during this period later tested positive for SARS-CoV-2. At the data cutoff point, >4 other household members who had never been to the worship hall also tested positive. Details of the demographic and epidemiologic information on the cases and clusters are provided in Appendix 1 Tables 2–8.

Consensus genomes of all 50 cases were constructed based on nanopore sequencing and refined by Illumina sequencing. On average, 62,387 reads/genome were obtained with 550× coverage for nanopore, and 18,747 reads/genome were obtained with 132× coverage for Illumina platform. The consensus genome size was ≈29.9 kbp with GC content ≈38%. The genomes were highly conserved with the first SARS-CoV-2 genome and had an average sequence identity of 99.98% (range 99.94%–100.0%). We identified 64 nonsynonymous substitutions from all 50 genomes (Appendix 1 Table 9). *Orf3a*-G251V was the most frequent amino acid substitution; 44/50 (88.0%) of the samples harbored this mutation, after which *Orf1ab*-H3233Y (30/50, 60.0%) and *S-*L8V (27/50, 54.0%) were most common.

Genomewide single-nucleotide polymorphisms were used to contextualize phylogenetic placement of Hong Kong strains in SARS-CoV-2 global phylogeny (Appendix 2 Figure 1). However, because the samples were taken at the early stage of global outbreak, the genetic variability between strains was limited, resulting in several unresolved branches and marginal supporting bootstrap values. Nevertheless, when compared with SARS-CoV-2 strains isolated from other regions, Hong Kong strains tended to aggregate mainly in 2 lineages. Lineage 1 consisted of 4 Hong Kong strains that clustered with most isolates from China (n = 32). Lineage 2, which consisted of 44 Hong Kong strains, was more closely related to strains seen in South Korea (n = 7) and France (n = 5).

**Figure 2:**
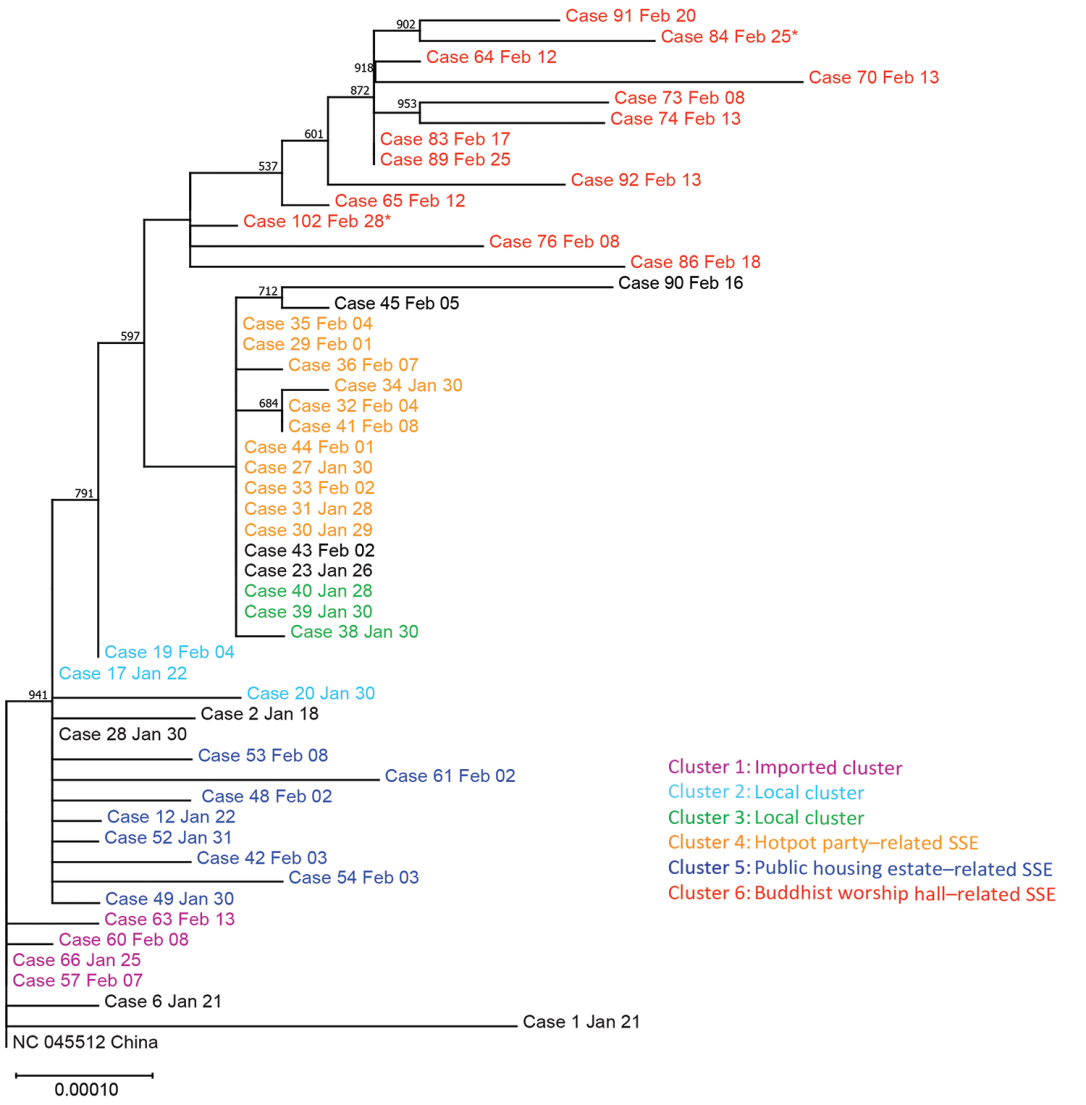
Maximum-likelihood phylogenetic tree of 50 coronavirus disease cases, Hong Kong, February 2020. The tree was rooted on the earliest published genome of severe acute respiratory syndrome coronavirus 2 (GenBank accession no. NC_045512.2). Bootstrap value was set at 1,000× and nodes with bootstrap value >50% were shown. Branch lengths were measured in number of substitutions per site. Samples are color-coded by epidemiologic link. Cases 84 and 102 were asymptomatic at the time of sample collection and are marked with asterisks. Each case is identified by case number used by the Centre of Health Protection, Department of Health, Hong Kong, and date of symptom onset. SSE, superspreading event.

In examining the phylogeny of the COVID-19 outbreak in Hong Kong, we identified 2 distinctive groups (Figure 2). The first group consisted of 2 imported cases and the cases in cluster 1. The second group originated with a single robust node with bootstrap value of 94% and a common mutation *Orf3a-*G251V. The second group could be further separated into 3 subgroups. The first subgroup mainly consisted of the cases in cluster 5, the public housing estate–related SSE. The second subgroup included cases in cluster 4 associated with the family hotpot party, cluster 3, and 2 isolated cases (case 23 and case 43). These samples shared the same missense mutations at *S*-L8V and *Orf1ab-*H3233Y. Finally, the third subgroup included the cases from cluster 6, an SSE originating from a Buddhist worship hall, in which *Orf1ab*-G295V were identified.

According to Bayesian time-scaled phylodynamic analysis, strict clock and relaxed clock models estimated the time of most recent common ancestor of COVID-19 outbreak in Hong Kong as December 24, 2019 (95% Bayesian credible interval [BCI] December 11, 2019–January 5, 2020). The evolutionary rate was 3.04 × 10^−3^ substitutions/site/year (95% BCI 2.04–4.09 × 10^−3^ substitutions/site/year) (Appendix 2 Figure 2). Based on demographic data, the average time from symptom onset to hospital admission was ≈8.5 days. The estimated reproduction number was calculated at 1.84 (95% BCI 1.37–2.35).

## Discussion

This study provides a territorywide overview of early COVID-19 outbreak in Hong Kong, an international city with borders connecting to mainland China, by integrating demographic, clinical, epidemiologic, phylogenomic, and phylodynamic data. In Hong Kong, most cases recorded in January 2020 were imported cases. After February 1, most were local cases and close contacts of those case-patients, indicating local community transmissions. Transmission in closed settings, especially during family and religious gatherings, is a hallmark of recent cases recorded in Hong Kong. Among 6 clusters identified on the basis of epidemiologic links, 3 (clusters 4–6; Figure 1) were considered SSEs because of the larger number of persons involved (n = 8–13). We performed whole-genome sequencing on all 50 cases to investigate phylogenetic relationship and transmission link.

The SARS-CoV-2 samples in Hong Kong had 99.98% identity to the reference genome (GenBank accession no. NC_045512.2) and demonstrated no apparent major genome modification since the initial COVID-19 outbreak in Wuhan. As shown in global phylogeny, SARS-CoV-2 genomes isolated in Hong Kong could be segregated into 2 lineages. Lineage 1 was phylogenetically related to the strains isolated from China and was the cause of the cases in Cluster 1. Lineage 2 was more closely related to strains from France and South Korea. It also harbored a common mutation at *Orf3a-*G251V, which accounted for 88.0% of cases in this study.

Regarding the local phylogenetic analysis, clustering of samples was highly concordant to the epidemiologic link, despite the marginally supportive bootstrap value of the nodes because of the limited genetic variability. Cluster 1 demonstrated the closest genetic distance to the reference genome among all cases reported in Hong Kong (Figures 1, 2). The index case of cluster 1 (case 66) was initially defined as possible local infection because the patient traveled to Guangdong Province, which was not considered to have active community transmission at that time. However, our sequencing result demonstrated that the genome of case 66 was 100% identical to the first published SARS-CoV-2 genome, and all cases in cluster 1 did not harbor *Orf3a*-G251V, which was recognized as a hallmark of local cases with unknown source in our community. Therefore, instead of possible local infections, cluster 1 was more likely imported from mainland china through index case-patient 66.

Cluster 5 originated within a public housing estate, in which a family of 3 members (cases 42, 48, and 49) were suspected to have been infected through a confirmed case-patient (case 12) who lived 10 stories above them in the same building, through a potentially faulty sewage pipe setup or other environmental exposure. Based on phylogenetic analysis, viral genomes in cluster 5 shared a similar genetic distance from the reference genome and were assigned to the same branch of the tree. This finding supports a potential transmission link among these cases.

**Figure 3:**
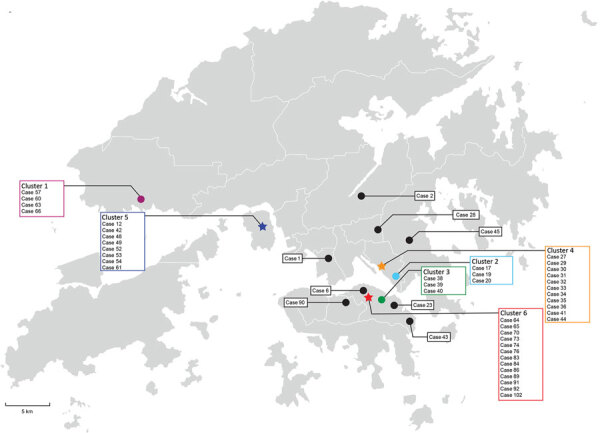
Geographic distribution of 50 coronavirus disease cases, Hong Kong, February 2020**.** Geographic information is marked according to the residence of the index case-patient in each cluster. Clusters known to be caused by superspreading events are marked by asterisks; other clusters are marked by dots.

Cluster 4 was a family gathering–associated SSE during Chinese New Year. In concert with epidemiologic information, all 11 cases from cluster 4 shared 3 common missense mutations, namely *S*-L8V, *Orf1ab*-H3233Y, and *Orf3a*-G251V; 7 cases shared identical genomes. Considering the fast-evolving property of RNA viruses, the presence of identical genetic sequences among the strains implies that transmission occurred over a short period or even in a single event. Meanwhile, 2 isolated cases (case 23 and case 43) and 3 cases from another local cluster (case 38, case 39, and case 40) shared highly similar genomes to those of cluster 4 (Figures 1, 2). Although no apparent epidemiologic links were observed, the high degree of genomic similarity suggests that these cases might have originated in a single source. That speculation was further supported by the geographic distribution of case-patients who lived near one another and whose social circles might have overlapped (Figure 3). Our results demonstrate that the integration of epidemiologic and phylogenetic data is critical for providing more accurate information about transmission patterns.

Cluster 6 was an SSE occurring in a Buddhist worship hall. Two missense mutations, *Orf1ab*-G295V and *Orf1ab*-L3606F, were unique to this cluster. Epidemiologic investigation identified a 43-year-old monk (case 102; Figure 1), who was the abbot of the worship hall and had traveled to mainland China in early January. He was sent to a quarantine center in late February after being linked to a series of confirmed cases connected to the worship hall. He was asymptomatic throughout the study period. Phylogenetic analysis showed that this case was closest to the root of the cluster (Figure 2), suggesting that case 102 could be the index patient of cluster 6. By the time of data cutoff, the cluster involved 13 patients and spread was ongoing. This pattern demonstrates the possibility of a hidden spreader as a source of COVID-19 community outbreak. That likelihood also highlights the importance of rapid quarantine of close contacts of confirmed case-patients, regardless of the presence of symptoms, to halt community spread.

In the evolutionary clock study, the reproduction number of COVID-19 within Hong Kong as of February 28, 2020, was estimated at 1.84 (95% BCI 1.37–2.35). That value strongly indicated that the outbreak in Hong Kong was ongoing, but it was smaller than the estimated reproduction number of 2.6 in Wuhan (*23*,*24*). The smaller value is a combined outcome of reduced growth rate and increased δ. The reduced growth rate is attributed to strong public health awareness among the general public, which resulted in greatly reduced social activities and strong compliance with mask-wearing during this period (*25*,*26*). The increased δ can be attributed to robust laboratory surveillance and fast quarantine time. In addition, time of most recent common ancestor for the cases in Hong Kong was determined to be December 24, 2019, ≈25 days before the first patient in our cohort (Case 2) demonstrated symptoms on January 18, 2020 (Figure 1).

Our study has several limitations. Although we included 53.8% of the cases reported in Hong Kong as of February 28, another 43 cases, including 2 fatal cases, were not analyzed in this study. Moreover, incubation periods of cases in which the source of infection is unknown might vary widely. Studies have demonstrated that incubation periods can vary from 4.5 to 15.8 days (*24*) and can be longer for patients experiencing mild symptoms. However, because patients might already be infectious during the incubation period, the reproductive number in this study could be underestimated. Furthermore, our calculations were based solely on phylodynamic analysis, which could differ from calculations on the basis of epidemiologic models. Finally, ambiguous bases were observed in some of our consensus genomes. This ambiguity is mainly because whole-genome sequencing was performed on respiratory specimens instead of viral culture, in which viral load plays a critical role in the subsequent genome quality as reflected by the cycle threshold of each specimen. The paucity of viral load in specimens could affect the yield of sequencing libraries. In our study, specimens with cycle threshold <28 were usually free of ambiguous bases. Nevertheless, the uncovered area only accounted for ≈1–3% of the entire viral genome, although the remaining mapped regions had an average coverage of >100×, which should provide sufficient and accurate information for subsequent analyses (Appendix 1 Table 10).

In conclusion, phylogenomic data were consistent with epidemiologic findings that transmission in closed settings, especially during family and religious gatherings, is a hallmark of COVID-19 outbreak in Hong Kong. Social distancing and vigilant infection control measures, such as rapid isolation of suspected or confirmed case-patients and their close contacts, are crucial for containing COVID-19 in the community.

Appendix 1 Additional information on territory-wide study of early coronavirus disease outbreak, Hong Kong, China.

Appendix 2 Supplemental methods on territory-wide study of early coronavirus disease outbreak, Hong Kong, China.
