# Supplementary material for: Territorywide Study of Early Coronavirus Disease Outbreak, Hong Kong, China
Source: Emerg Infect Dis. 2021 Jan;27(1):196–204. doi: 10.3201/eid2701.201543 (PMC7774584; doi:10.3201/eid2701.201543)
Supplement: Appendix 2 — Supplemental methods on territory-wide study of early coronavirus disease outbreak, Hong Kong, China. [file 20-1543-Techapp-s2.pdf]

Article DOI: https://doi.org/10.3201/eid2701.201543

Territory-Wide Study of Early Coronavirus Disease Outbreak, Hong Kong, China

Appendix 2

Supplemental Methods

Case Definitions

Imported cases referred to case-patients who had records of traveling to Wuhan City or Hubei Province, China ≤14 days before symptom onset. Possible local cases referred to case-patients who had records of traveling to other regions of China or other countries where active community transmission was not confirmed at the time of the respective case. Local cases referred to case-patients who did not have a travel history ≤14 days before symptom onset.

Amplification of SARS-CoV-2 Genome using Multiplex PCR

The viral cDNA was amplified by using 2 PCRs containing tiled, multiplexed primers as described in the ARTIC network (*1*). These primers (n = 196) generated overlapping 400-bp amplicons across the entire genome of COVID-19 (accession no. NC_045512). Each PCR contained a primer pool of 98 primers (Appendix 1 Table 1, https://wwwnc.cdc.gov/EID/article/27/1/20-1543-App1.xlsx). Eventually, the PCR mastermix (50µL) included 2·5µL of cDNA, 5·0µL of 5X Q5 Reaction Buffer, 0·5µL of 10mM dNTP mix, 0·25µL of Q5 Hot Start DNA Polymerase (New England Biolabs, https://www.neb.com/) and 3·6µL of 10µM primer pool 1 or 2, and 13·15µL of nuclease-free water. The mixtures were incubated at 98°C for 30 s, followed by 35 cycles at 98°C for 15 s and 65°C for 5 min. The PCR amplicons were then purified by 1X Agencourt AMPure XP beads (Beckman Coulter, https://www.beckmancoulter.com/).

Bioinformatic analysis of Nanopore Sequencing Data

Nanopore sequencing data were analyzed using a protocol modified from Artic Network nCoV-2019 novel coronavirus bioinformatics protocol (*2*). In brief, sequencing results obtained from Nanopore sequencing were first based-called using guppy-basecaller (v3.4.5). The

sequencing reads were then demultiplexed by Porechop (v0.2.4) to correct the significant barcoding misassignments and mapped against the SARS-CoV-2 reference genome Wuhan-Hu-1 (accession no. NC_045512) using BWA (v0.7.17) and Samtools (v1.7). The BAM file was used for variant calling by Medaka (v0.11.5) with threshold value set at 1 to ensure haploid decoding. To evaluate the performance of variant calling by medaka, variants were validated again using Nanopolish (v0.11.0) with haploid decoding, and candidate variants from the aligned reads were extracted when the variant frequency was >40%. The consensus genome was assembled based on the VCF from Medaka (v0.11.5) and BAM file. Depth per base required for variant calling was set at >20X with minimum required base Phred score of 10.

Modified Artic Network nCoV-2019 novel coronavirus bioinformatic protocol

porechop -i [$fastq_file] -o [$output_file] -t [$thread no.]

source activate artic-ncov2019

artic minion–normalize 200–threads [$thread no.]–scheme-directory ~/artic-ncov2019/artic-ncov2019/primer_schemes–read-file [$porechop_output] nCov-2019/V1 [$filename]–skip-nanopolish

source ~/.bashrc

source activate medaka #medaka v0.11.5

medaka consensus [$filename.primertrimmed.sorted.bam] [$filename.hdf]

medaka snp /home/gilman_siu/artic-ncov2019/artic-ncov2019/primer_schemes/nCov-2019/V1/nCov-2019.reference.fasta [$filename.hdf] [$filename.primertrimmed.sorted.bam] [$filename.primertrimmed.medaka.vcf]

source ~/.bashrc

source activate artic-ncov2019

margin_cons_medaka–depth 20–quality 10 /home/gilman_siu/artic-ncov2019/artic-ncov2019/primer_schemes/nCov-2019/V1/nCov-2019.reference.fasta [$filename.primertrimmed.medaka.vcf] [$filename.primertrimmed.sorted.bam] >[$filename.consensus.fasta]

References:

1. Quick J. Artic Network-nCoV 2019 sequencing protocol. 2020 [cited 2020 Feb 24]. https://artic.network/ncov-2019

2. Loman N, Rambaut A. nCoV-2019 novel coronavirus bioinformatics protocol. 2020 [cited 2020 Mar 10]. https://artic.network/ncov-2019/ncov2019-bioinformatics-sop.html

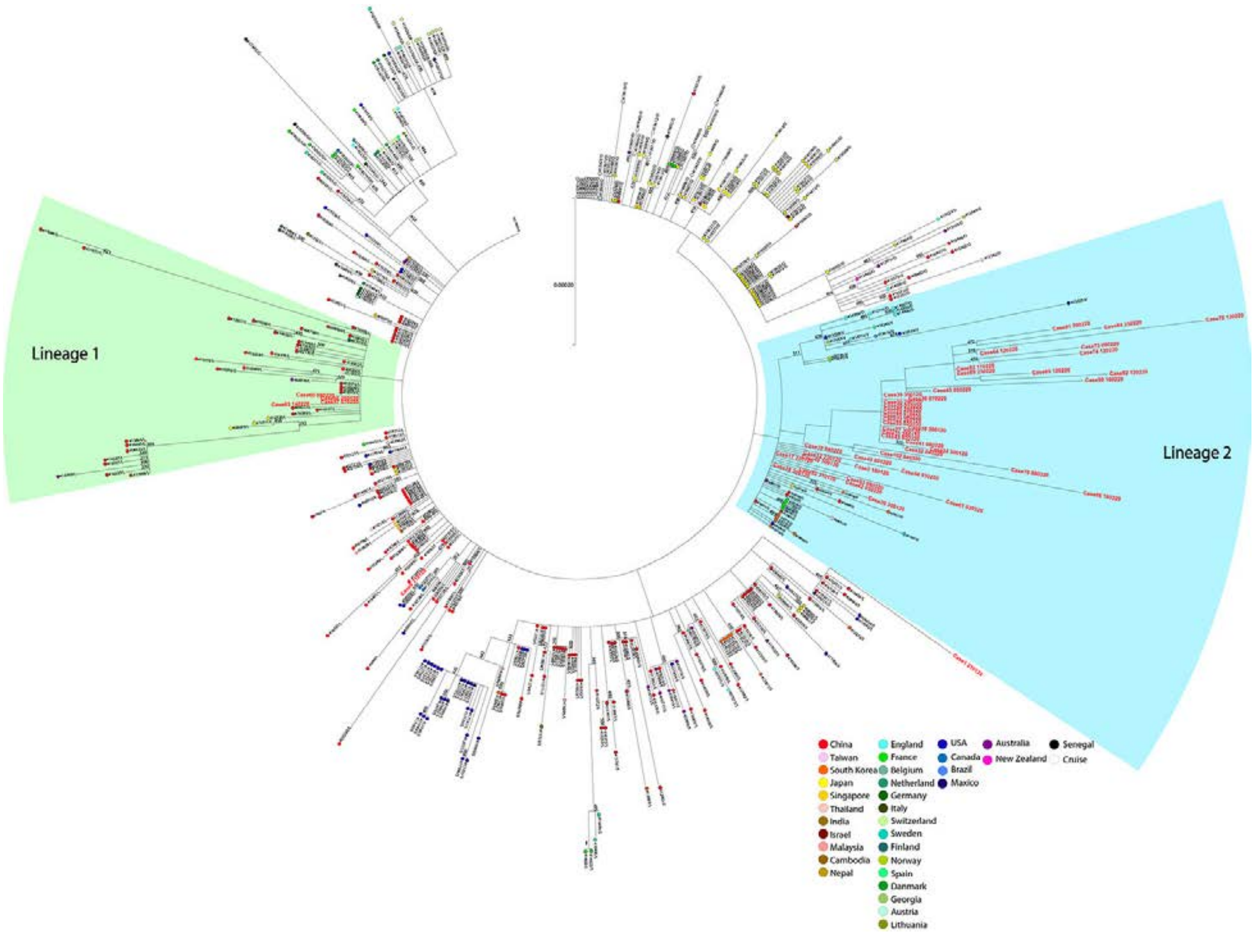

Appendix 2 Figure 1. Maximum-likelihood phylogenetic tree constructed based on 50 severe acute respiratory syndrome coronavirus 2 genomes in Hong Kong, February 2020, and genomes collected from GISAID datahub. A total of 478 worldwide genomes as of February 28, 2020 were available from GISAID Global Cases COVID-19 database. All genomes were downloaded and the phylogenetic tree was built by using Maximum Likelihood with bootstrap value set and rooted on the earliest published genome of severe acute respiratory syndrome coronavirus 2 (GenBank accession no. NC_045512.2). Bootstrap value was set at 500× and nodes with bootstrap value >50% were shown. Branch lengths were measured in number of substitutions/site. All Hong Kong strains are highlighted in red. Hong Kong strains showed limited genetic variability and tended to aggregate in two lineages, highlighted in green and cyan.

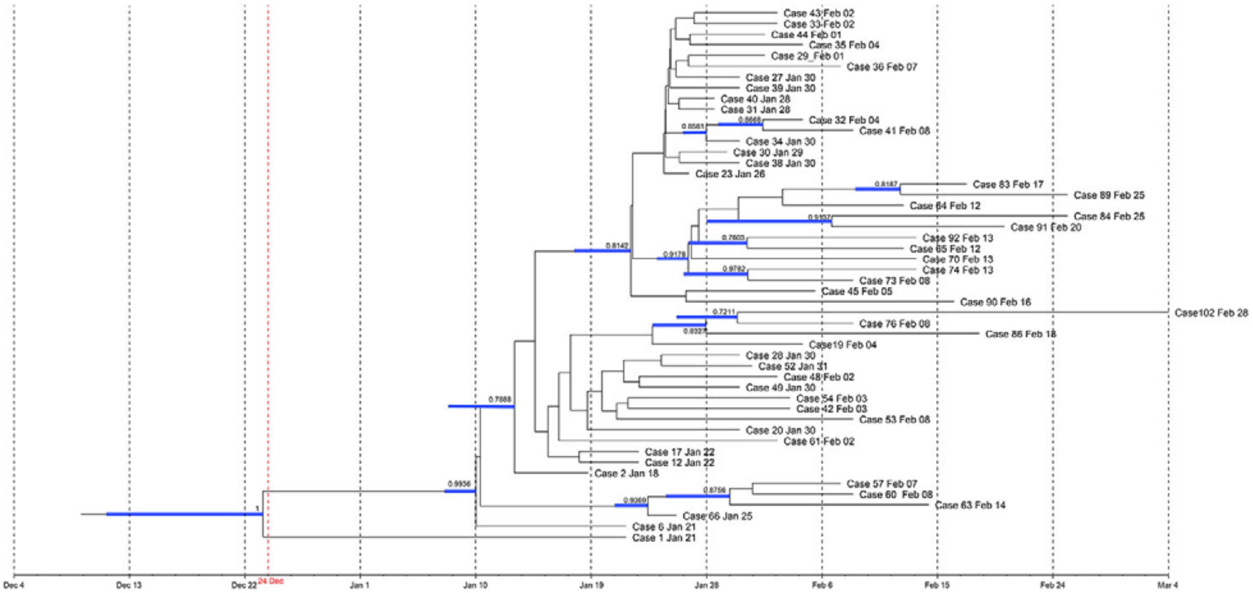

Appendix 2 Figure 2. Bayesian maximum clade creditability time-scaled phylogeny of the early COVID-19 outbreak in Hong Kong. Bayesian time-scaled phylogeny plotted using 50 COVID-19 genomes collected in this study. The analysis was undertaken using GTR+G+I model, coalescent exponential population, strict clock setting, sampling 100,000 trees from 1 billion generations. Uncertainty for the date of each node (95% highest posterior density intervals) is displayed in blue bars. Nodes with posterior higher than 70% were displayed.
